# Utilization, satisfaction, and barriers to antenatal care among pregnant women in Gadarif State during the Sudan war: a cross-sectional study

**DOI:** 10.1186/s12884-025-07556-6

**Published:** 2025-04-11

**Authors:** Eithar M. Ali, Ahmed Balla M. Ahmed, Muhannad Bushra Masaad Ahmed, Alaa T. Omer, Elshaima Ibrahim Mohamedelhassan Ahmedtaha, Khadija A. Khalil, Logien Abuelgasim Ahmad Ibrahim, Maab Hassan Ahmed Abdallah, Mohamed Osman Mohamed Abdelrahim, Sakhr Hassan Ahmed Abdallah, Sohaib Mohammed Mokhtar Ahmed

**Affiliations:** 1 Educational Development Center, University of Khartoum, Khartoum, Sudan; 2 Present Address: Faculty of Medicine, University of Khartoum, Al-Qasr Street, PO Box: 102, Khartoum, 11111 Sudan; 3 Faculty of Medicine and Health Sciences, University of Gadarif, Gadarif, Sudan

**Keywords:** Utilization, Satisfaction, Barriers, Antenatal care, Sudan, War

## Abstract

**Background:**

Healthcare services during wartime face significant challenges. Pregnant women seeking antenatal care (ANC) during the Sudan conflict encounter numerous barriers, impacting their access and satisfaction. This study assessed ANC utilization, satisfaction, and barriers faced by pregnant women in Gadarif State during the ongoing war.

**Methods:**

This cross-sectional study was conducted at Gadarif Hospital for Obstetrics and Gynecology from September to November 2024, targeting women who had given birth at the hospital. A questionnaire with four sections assessed utilization, satisfaction, and barriers with ANC during wartime. Cross-tabulations and chi-square tests analyzed associations between categorical variables, with *p*-values < 0.05 considered significant.

**Results:**

A total of 345 pregnant women participated in this study. ANC utilization was reflected in a median of 4.0 visits (IQR = 2.0), with the first visit occurring at a median of 13 weeks (IQR = 13). Overall, 90.4% of participants reported being satisfied with ANC services, although satisfaction with interpersonal communication skills was the lowest at 6.4%. Barriers to access included lack of privacy (33.3%) and irregular facility operating hours (21.4%), while the primary reasons for missing ANC visits were transport challenges and the absence of night-duty staff.

**Conclusion:**

This study revealed a surprisingly high level of satisfaction with ANC services, despite key barriers such as lack of privacy and irregular health facility operating hours. While ANC attendance was relatively good, visits tended to start later than recommended. Interventions should focus on addressing these barriers by improving service accessibility and ensuring consistent care. Enhancing early engagement with ANC is also critical to improving maternal health outcomes.

**Supplementary Information:**

The online version contains supplementary material available at 10.1186/s12884-025-07556-6.

## Background

Antenatal care, or prenatal care, refers to the medical support and services offered throughout pregnancy, from its onset until delivery [1]. This period is vital for implementing interventions that promote the health of both mother and baby, with at least four visits significantly improving access to essential maternal healthcare [2]. Previous studies have emphasized numerous challenges faced by pregnant women in conflict-affected areas, including limited access to healthcare facilities, inadequate medical supplies, and heightened exposure to violence [3, 4]. All these factors significantly limit their access to essential maternal healthcare services, potentially resulting in higher maternal and infant mortality rates, as well as increased complications during pregnancy and childbirth [4].

The war not only restricts access to care but also undermines patient satisfaction with the services available. Patient satisfaction reflects the extent to which individuals are content with their healthcare experiences, encompassing technical, interpersonal, and organizational aspects. In the context of antenatal care (ANC), satisfaction with the quality of care received and a patient’s willingness to return to or recommend a facility are critical [5].

Since April 15, 2023, the war in Sudan has inflicted immense suffering, marked by widespread violence, displacement, and a deepening humanitarian crisis impacting millions [6, 7]. Over 100 attacks on healthcare facilities have been recorded since the conflict began, and more than 30% of public hospitals have ceased operations within the first year of the war [8, 9]. In addition, access to quality antenatal care, safe delivery facilities, skilled healthcare providers, and essential obstetric services has been gravely undermined due to disruptions caused by the conflict [10].

Gadarif State is among the most severely affected regions in Sudan, facing a massive influx of displaced people. As conflict forces populations from neighboring areas into Gadarif, the state grapples with overcrowding, resource shortages, and escalating humanitarian demands, exacerbating the crisis more than in other regions [11]. These unique circumstances make Gadarif a critical focus for understanding how antenatal care services are being utilized, the barriers faced by pregnant women, and their satisfaction with the care they receive amid the ongoing war. Building on these challenges, this study aimed to explore the utilization and satisfaction with antenatal care (ANC) services, as well as the barriers faced by pregnant women at Gadarif Hospital for Obstetrics and Gynecology during the 2023 Sudan conflict.

## Methods

### Study design and setting

This cross-sectional study was conducted from September to November 2024 among pregnant women receiving antenatal care at Gadarif Hospital for Obstetrics and Gynecology. The study’s methodology and findings are described in detail in the manuscript, adhering to the STROBE (Strengthening the Reporting of Observational Studies in Epidemiology) guidelines [12].

### Study population

This study included pregnant women who had completed their pregnancies and given birth at Gadarif Hospital for Obstetrics and Gynecology, ensuring they had fully experienced the pregnancy period and had the opportunity to utilize ANC services throughout. Gadarif Hospital for Obstetrics and Gynecology is the largest public tertiary hospital in Gadarif State, eastern Sudan. Located in Gadarif city, the state capital, the hospital provides comprehensive maternity care, including antenatal, delivery, and postnatal services, to all women in eastern Sudan, regardless of their residency (urban or rural) or pregnancy status (complicated or uncomplicated). Annually, approximately 8,000 newborns are delivered at the hospital [13]. The hospital’s medical staff includes consultants and specialists in obstetrics and gynecology, along with registrars, general practitioners, house officers, nurses, and laboratory technicians. Additionally, the hospital serves as a teaching institution in collaboration with the Faculty of Medicine, University of Gadarif.

We excluded critically ill pregnant women and those who did not consent to participate in the study.

### Sampling

The minimum sample size, calculated using the Cochran formula [14], was 384 participants. This approach was used due to the lack of official records. The calculation was based on a 95% confidence interval (CI), a 5% margin of error, and an expected frequency of 50%, with the target population (N) being unknown. Convenience sampling was used due to the practical challenges of accessing a comprehensive population list and the need to select participants from those readily available at Gadarif Hospital. Data were collected from women who had completed their pregnancies and delivered their babies to ensure they had fully experienced the pregnancy period and had the opportunity to utilize ANC services throughout.

### Data collection tool

A face-to-face interview questionnaire was used in this study (Additional file 1). The questionnaire was developed based on a literature review and included both original and adapted components. The questionnaire was divided into four sections. The first section, designed by the authors, gathered sociodemographic and maternal health characteristics of the participants. The second section assessed satisfaction levels with antenatal care, based on a study conducted in Addis Ababa, Ethiopia [15]. The third section assessed barriers to using antenatal care, adapted from a study in Ethiopia [16]. The fourth section included a single question about the reasons for not attending antenatal care visits. The questionnaire was reviewed by an expert in obstetrics and gynecology and piloted among women who had completed their pregnancies and delivered. Participants in the pilot phase were excluded from the final sample.

### Outcome variables

Utilization of ANC is defined by early attendance, where the first ANC visit occurs within the first trimester before 12 weeks, and adequate follow-up, with a minimum of four ANC visits throughout pregnancy [17].

Antenatal care service satisfaction refers to the extent to which a woman receiving care feels content with the quality and experience of the antenatal services provided to her [18]. We measured overall satisfaction with ANC services, categorized as satisfied or unsatisfied. Additionally, we assessed satisfaction with specific aspects of care, including respectful treatment, confidentiality, interpersonal communication, waiting time, appointment availability, counseling on pregnancy complications, birth planning advice, and perceived provider competence, all classified as either satisfied or unsatisfied.

### Independent variables

The independent variables in this study include age, age at first birth, residence, level of education, distance from a health facility, number of pregnancies, occupation, socio-economic status, age of last child, number of babies delivered, number of family members living in the same house, ANC provider, source of information on maternal healthcare services, and primary reasons participants faced challenges accessing healthcare services.

Barriers to ANC visits refer to the factors that hinder or limit pregnant women’s ability to access and utilize antenatal care services effectively. These barriers include:

1. Structural barriers which include:
  Lack of privacy during ANC follow-up, favoritism in health services, and irregular opening of health facilities/convenient service times.
2. Security-related barriers which include: Restricted movement due to insecurity and displacement far from health facilities.
3. Health system barriers which include: Fleeing of local health providers, looting or disruption of medical supplies, and destruction/shutdown of health facilities.

### Data analysis and management

Data were analyzed using descriptive and inferential statistical methods. Descriptive statistics summarized demographic and antenatal care (ANC) variables, including median, interquartile range (IQR), and percentages, as appropriate. Pearson’s correlation was utilized to examine relationships between demographic factors and ANC utilization, with significance levels set at *p* < 0.05. Cross-tabulations and chi-square tests were conducted to assess associations between categorical variables such as residence and satisfaction levels with ANC services. All statistical analyses were performed using Jamovi version 2.6.19, ensuring proper data handling and integrity. Missing data were managed using listwise deletion to maintain analytical consistency.

## Results

The study achieved a 90% response rate among the 345 participants who completed the interview questionnaire. The 10% non-response was due to refusals to participate, early withdrawal from the interview, or incomplete responses. The median age was 28 years (IQR=10), with a median age at first birth of 21 years (IQR=5). Most participants resided in urban areas (60.9%), with secondary education being the most common level attained (35.1%). Socio-economic status was fairly distributed, with 37.6% earning 200,000–300,000 Sudanese pounds (94.9–142 U.S dollars). Healthcare access varied, with 59.7% living near a health facility, and 95.9% receiving antenatal care (ANC) from doctors. Family size median was 7.0 members (IQR = 3.0), and 53.3% reported unplanned pregnancies. Housewives constituted the majority (72.7%), and healthcare providers were the primary source of maternal health information (93.9%). When assessing utilization of ANC, participants had a median of 4.0 ANC visits (IQR = 2.0), with the first visit starting at a median of 13 weeks (IQR=13) **(**Table 1**)**.

**Table 1:** Sociodemographic, maternal health characteristics, and utilization of ANC services among the study population

	Overall (*N* = 345)
**Age**	
Median (IQR)	28 (10)
**Age at first birth**	
Median (IQR)	21 (5)
**Residence**	
Rural	135 (39.1%)
Urban	210 (60.9%)
**Level of Education**	
No education	61 (17.7%)
Primary	58 (16.8%)
Secondary	121 (35.1%)
Higher	100 (29.0%)
Informal education	5 (1.4%)
**Distance from a health facility**	
Far	139 (40.3%)
Near	206 (59.7%)
**Number of Pregnancies**	
Median (IQR)	3 (2)
**Planned Pregnancy**	
No	184 (53.3%)
Yes	161 (46.7%)
**Occupation**	
Governmental employee	44 (12.8%)
Housewife	251 (72.7%)
Private business	16 (4.6%)
Retired	1 (0.3%)
Self-employed	10 (2.9%)
Student	10 (2.9%)
Unemployed	13 (3.8%)
**Socio-economic Status**	
Less than 200,000 Sudanese pounds (less than 94.9 U.S dollars)	92 (26.7%)
200,000–300,000 Sudanese pounds (94.9–142 U.S dollars)	130 (37.6%)
More than 300,000 Sudanese pounds (more than 142 U.S dollars)	123 (35.7%)
**Age of last child**	
Median (IQR)	2.0 (1.0)
**Number of babies delivered**	
Median (IQR)	3.0 (2.0)
**Number of family members living in the same house**	
Median (IQR)	7.0 (3.0)
**ANC provider**	
Doctor	331 (95.9%)
Midwife	12 (3.5%)
Others	2 (0.6%)
**Source of Information on MHCS**	
Health care provider	324 (93.9%)
Others	8 (2.3%)
Social media	13 (3.8%)
**Number of ANC visit**	
Median (IQR)	4.0 (2.0)
**ANC starting time in weeks**	
Median (IQR)	13 (13)

Table 2 presents participants’ levels of satisfaction with various aspects of antenatal care (ANC). Overall satisfaction with ANC services was high, with 90.4% expressing satisfaction. Advice on complications during pregnancy (93.9%) and patients’ views on providers’ competence (97.4%) were the most positively rated aspects. However, interpersonal communication skills showed the lowest satisfaction (6.4%), followed by waiting time, with 88.7% reporting dissatisfaction. Confidentiality and advice on birth plans were moderate concerns, with 29.3% and 35.4% unsatisfied, respectively.

**Table 2:** Satisfaction levels with antenatal care services

Aspect	Unsatisfied	Satisfied
**Satisfaction with ANC service**	33 (9.6%)	312 (90.4%)
**Providing respectful care**	37 (10.7%)	308 (89.3%)
**Providing confidential care**	101 (29.3%)	239 (70.7%)
**Interpersonal communication skill**	323 (93.6%)	22 (6.4%)
**Waiting time**	306 (88.7%)	39 (11.3%)
**Appointment availability**	55 (15.9%)	290 (84.1%)
**Advice on complications during pregnancy**	21 (6.1%)	324 (93.9%)
**Advice on birth plan**	122 (35.4%)	223 (64.6%)
**Patients’ views on provider’s competence**	9 (2.6%)	336 (97.4%)

Table 3 highlights barriers to accessing antenatal care (ANC) among participants. The most frequently reported problem was a lack of privacy during ANC follow-up, affecting 33.3% of respondents. Other challenges included irregular opening times of health facilities (21.4%), displacement far from health services (14.2%), and favoritism in the provision of healthcare (13.6%). Insecurity-related issues, such as restricted movement (11.6%) and the fleeing of local health providers (16.2%), were also noted. Additionally, 16.2% reported disruptions in medical supplies, while 14.5% faced facility destruction or shutdowns.

**Table 3:** Barriers to accessing healthcare services

Problem	No	Yes
**a. Lack of privacy during ANC follow-up**	230 (66.7%)	115 (33.3%)
**b. Favoritism in health services**	298 (86.4%)	47 (13.6%)
**c. Irregular opening of health facilities/Convenient service times**	271 (78.6%)	74 (21.4%)
**d. Restricted movement due to insecurity**	305 (88.4%)	40 (11.6%)
**e. Displacement far from health facilities**	296 (85.8%)	49 (14.2%)
**f. Fleeing of local health providers**	289 (83.8%)	56 (16.2%)
**g. Looting or disruption of medical supplies**	289 (83.8%)	56 (16.2%)
**h. Destruction/Shutdown of health facilities**	295 (85.5%)	50 (14.5%)

**Fig. 1:**
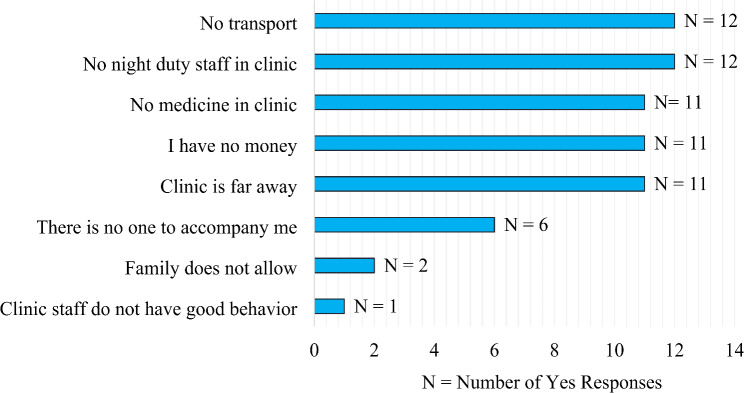
Reasons for not attending ANC visit

Figure 1 illustrates the primary reasons participants faced challenges in accessing healthcare services. Lack of transport and the absence of night-duty staff in clinics were the most common issues, each reported by 12 participants. Additionally, 11 participants cited the lack of medicine, financial constraints, and long distances to clinics as barriers. Six participants mentioned the absence of someone to accompany them, while fewer respondents reported familial disapproval (2 participants) or poor behavior from clinic staff (1 participant).

Table 4 presents the relationships between various demographic and antenatal care (ANC) variables using Pearson’s correlation analysis. A weak negative correlation was observed between age and the number of ANC visits (*r* = -0.110, *p* = 0.042), suggesting that older women attended fewer ANC sessions. Similarly, the number of pregnancies and the number of household family members were negatively associated with ANC visits (*r* = -0.163, *p* = 0.002 and *r* = -0.184, *p* < 0.001, respectively). Conversely, a weak positive correlation was found between age at first birth and ANC initiation timing (*r* = 0.122, *p* = 0.024), indicating that women who had their first child at a later age began ANC earlier. Notably, earlier ANC initiation was strongly correlated with a higher number of sessions attended (*r* = 0.300, *p* < 0.001).

**Table 4:** Correlations between demographic variables and antenatal care utilization

Variable 1	Variable 2	Pearson’s *r*	*p*-value	Interpretation
Age	Number of ANC visits	−0.110	0.042	Older pregnant women tended to have fewer ANC visits
Age at first birth	ANC starting time in weeks	0.122	0.024	Women who had their first child later in life were more likely to start ANC earlier during pregnancy.
Number of pregnancies	Number of ANC visits	−0.163	0.002	Women with more pregnancies tended to utilize ANC services less frequently.
Number of family members in the house	Number of ANC visits	−0.184	< 0.001	Larger households negatively affected ANC attendance.
ANC starting time in months	Number of ANC visits	0.300	< 0.001	Women who started ANC earlier tended to attend more ANC sessions.

We examined the influence of residence (rural vs. urban) on antenatal care (ANC) services. Urban residents reported more privacy issues during ANC follow-ups (**Χ²=4.44**, ***p* = 0.04**). Dissatisfaction with ANC services was significantly higher among rural residents (**Χ²=7.07**, ***p* = 0.01**). Urban residents showed better appointment adherence (**Χ²=11.96**, ***p* < 0.01**) and received more advice on pregnancy complications (**Χ²=16.42**, ***p* < 0.01**). Despite these disparities, satisfaction with respectful care remained high in both groups (**Χ²=1.58**, ***p* = 0.21**). Those living farther away from a health facility reported more unsatisfactory waiting times (**Χ²=18.14**, ***p* < 0.01**) and disruptions in medical supplies (**Χ²=5.07**, ***p* = 0.02**). However, they had similar satisfaction levels with respectful care (**Χ²=2.11**, ***p* = 0.15**) and healthcare providers’ professional skills (**Χ²=0.07**, *p*** = 0.80**) compared to those living nearer. Notably, near residents to a health facility exhibited better appointment adherence (**Χ²=10.57**, ***p* < 0.01**) and ANC visit consistency (**Χ²=6.70**, ***p* = 0.01**).

## Discussion

The ongoing Sudan war has severely disrupted the healthcare system, including antenatal care (ANC) services. Despite these challenges, no prior studies have assessed ANC services in Gadarif State during the current war. This study aimed to investigate pregnant women’s satisfaction with and utilization of ANC services, as well as the barriers they face during the Sudan conflict.

This study identified a notable delay in ANC utilization, with the first visit typically occurring at a median of 13 weeks, aligning with trends in other developing regions where ANC typically begins between 13 and 17 weeks into pregnancy [19]. The median number of pregnancies and ANC visits attended indicate a trend of multiparity and consistent ANC utilization, which is beneficial for monitoring maternal and fetal health.

The satisfaction level with ANC services, as perceived by patients, was remarkably high, exceeding the levels reported in Ethiopia [15]. However, this high satisfaction may not necessarily reflect a good quality of services but rather low patient expectations. In resource-limited settings or during crises, patients often adjust their expectations to match the available services, resulting in high satisfaction scores despite potential deficiencies in service quality.

On the other hand, dissatisfaction with specific aspects of care was notable. A high level of dissatisfaction was reported regarding waiting times, with almost one-third raising concerns about confidentiality and one-tenth expressing dissatisfaction with respectfulness. These findings highlight a disparity in the perceived quality of care, which could potentially influence women’s willingness to seek ANC services. Nonetheless, these levels are more favorable compared to findings from Ethiopia [15].

The least satisfactory aspect identified in this study was the interpersonal communication skills of providers. This aligns with findings from a study in Kenya, which reported inadequate communication as the leading cause of client dissatisfaction [20].

The study highlights several significant barriers to accessing ANC, encompassing logistical, operational, financial, and social challenges. Confidentiality concerns, reported by one-third of participants, reflect a lack of privacy during ANC follow-ups. Previous research consistently identifies confidentiality as a major barrier to accessing healthcare services among women seeking ANC [21]. Irregular opening hours and inconvenient service times, cited by one-fifth of participants, underscore the need for improved service delivery models. Insecurity further limited their ability to access healthcare facilities. A report by the International Federation of Red Cross and Red Crescent Societies (IFRC) emphasized that safety concerns during conflicts significantly hinder movement to health facilities, thereby affecting access to essential maternal care [22].

Additionally, displacement far from health facilities was reported as a critical access barrier by some respondents. A similar study conducted during the Sudan war found that displacement posed significant challenges for pregnant women during the conflict [10]. Research suggests that mobile health units or community outreach programs can effectively address the gaps caused by geographic displacement [23]. Furthermore, around one-sixth of participants indicated that the fleeing of local health providers was a barrier to receiving ANC, leading to a shortage of healthcare workers. This shortage has devastating effects on healthcare delivery and access, both during conflicts and in their aftermath. Finally, around one-sixth of respondents reported disruptions to medical supplies as a significant barrier to accessing ANC, a challenge that has been well-documented in the context of the current war [24].

The primary reasons for not attending ANC were lack of transport and the absence of night-duty staff in clinics. The lack of reliable transportation often results in missed appointments and delays in care, which can negatively impact both maternal and fetal health. Similar transportation challenges were highlighted in a study conducted in South Sudan [25]. Additionally, the unavailability of medicines and financial constraints were significant barriers to accessing ANC. A study from Somaliland suggested that reducing financial burdens by making services more affordable, or even free, could enhance ANC utilization [26].

A weak negative correlation between age and the number of ANC visits in this study suggests that older women may attend fewer sessions, potentially due to previous experiences or perceived health needs. However, a study in Africa found that older mothers were more likely to prioritize and regularly attend ANC visits [27]. Similarly, an Ethiopian study concluded that women aged 44–49 were 2.993 times more likely to utilize four or more ANC visits compared to those aged 15–19 [28].

In this study, women with more pregnancies and larger households were less likely to frequently utilize ANC services. This trend may reflect increased responsibilities or reliance on previous childbirth experiences, reducing their engagement with healthcare services. This finding aligns with an Ethiopian study where women who had birthed one child were 1.651 times more likely to utilize four or more ANC visits compared to those with no births, and women with two births were 2.875 times more likely to do so [28].

A positive correlation between the timing of the first ANC visit and the number of visits attended highlights the importance of early initiation of care in achieving better health outcomes. This observation is consistent with findings from a Tanzanian study [29].

Significant differences were observed between urban and rural residents regarding ANC experiences. Urban residents reported more privacy concerns during follow-ups but demonstrated better adherence to appointments and greater access to advice on pregnancy complications, similar to findings from a multi-country study [30]. In contrast, rural residents expressed higher dissatisfaction with ANC services, suggesting that urban settings may offer a more favorable environment for ANC utilization. This emphasizes the need for targeted interventions to improve service delivery in rural areas. Interestingly, an Ethiopian study from 2020 found that rural residency was significantly associated with higher satisfaction scores [31].

Despite these differences, satisfaction with respectful care was consistently high among both urban and rural participants. This is an encouraging finding, indicating that healthcare providers are generally perceived as respectful, fostering trust and encouraging women to seek care regardless of their location.

This study provides valuable insights into antenatal care utilization, satisfaction, and barriers in a conflict-affected setting. It is one of the few conducted in Sudan during the war, capturing real-time experiences of pregnant women. Including diverse socioeconomic and geographic backgrounds enhances generalizability within safer regions. By addressing multiple dimensions of antenatal care, the study contributes to evidence-based policy recommendations for improving maternal health services in crises.

This study has several limitations. The cross-sectional design limits causal inferences, and the use of non-probability convenience sampling may introduce bias. The study focused on pregnant women in safer areas, potentially excluding those in highly insecure regions. Additionally, the questionnaire was not standardized, and treating barriers and satisfaction as dichotomous variables may have restricted response depth. The study also does not comprehensively address maternal and neonatal outcomes or broader cultural and social influences on healthcare-seeking behaviors. Potential underreporting of sensitive barriers, such as domestic violence or stigma, could further impact findings.

## Conclusion

This study highlights the ANC services in Gadarif State during the ongoing Sudan war, shedding light on pregnant women’s experiences, barriers, and satisfaction with these services during a time of crisis. While the overall satisfaction with ANC services was remarkably high, this may reflect adjusted expectations rather than superior service quality. Despite adequate ANC utilization, delays in ANC initiation, along with major barriers such as irregular opening of health facilities and lack of privacy during ANC follow-up, were key findings.

Interventions should prioritize early ANC initiation by improving access to services in remote and insecure areas through mobile health units and community-based care. Strengthening the healthcare workforce by ensuring night-duty staff availability can enhance service accessibility. Interventions should focus on ensuring consistent and timely opening of health facilities to prevent delays in ANC initiation and enhancing privacy during ANC follow-up to improve the quality of care and satisfaction.

## Electronic supplementary material

Below is the link to the electronic supplementary material.

Supplementary Material 1

## Data Availability

“The datasets used and/or analysed during the current study are available from the corresponding author on reasonable request.”

## Abbreviations

ANC: Antenatal care
MHCS: Maternal health care services
IFRC: International Federation of Red Cross and Red Crescent Societies
