# Supplementary material for: Utilization, satisfaction, and barriers to antenatal care among pregnant women in Gadarif State during the Sudan war: a cross-sectional study
Source: BMC Pregnancy Childbirth. 2025 Apr 11;25:428. doi: 10.1186/s12884-025-07556-6 (PMC11992725; doi:10.1186/s12884-025-07556-6)
Supplement: Supplementary file 1 — Supplementary Material 1 [file 12884_2025_7556_MOESM1_ESM.docx]

**Utilization, satisfaction, and barriers to antenatal care among pregnant women in Gadarif State during the Sudan war: A cross-sectional study.**

The Questionnaire

1] Sociodemographic and Maternal Health Data:

**age**

…………….

**age at first birth**

…………………

**Place of residence**

- Urban
- Rural

**Level of education**

- No education
- Primary
- Secondary
- Higherl

**Distance from a health facility**

- Near
- Far

**Number of pregnancies**

**………………………..**

**Planned pregnancy**

- Yes
- No

**Occupation**

- Housewife
- Governmental employee
- Private business
- Self-employed
- Retired
- Unemployed
- Student

**Socio-economic status**

- Less than 200,000 SDGs
- 200,000 - 300,000 SDGs
- More than 300,000 SDGs

**Age of last child**

- 1 year
- >1 year

**Number of babies delivered**

- 1
- 2–5
- >5

**Number of family members living in the same house**

- <5
- 5–10
- >10

**Number of ANC visit**

● One

● Two

● Three

● ≥Four

**ANC starting time in months**

● Before 3 months

● 3 months

● 4 months

● 5 months

● After 6 months

**ANC provider**

● Doctor

● Midwife

● Others

**Source of Information**

**on MHCS**

- Health care provider
- Social Medias
- Others

2] Satisfaction levels with antenatal care services:

**Satisfaction with ANC service**

Satisfied

Unsatisfied

**Providing respectful care**

Yes

No

**Providing confidential care**

Yes

No

**Interpersonal communication skill**

Effective

Non-effective

**Waiting time**

<60minutes

60 minutes

**Appointment**

Yes

No

**Advise on complication during pregnancy**

Yes

No

**Advise on birth plan**

Yes

No

**Professional’s skill**

competent

Not-competent

3] Barriers to the use of antenatal care:

1. **Lack of privacy is a problem during ANC follow-up**

- Yes
- No

1. **Favoritism in the provision of health services on a factor** basis

- Yes
- No

1. **Irregular opening of health facilities/ Convenient time of service**

- Yes
- No

1. **Restricted movement to operational health facilities due to insecurity**

- Yes
- No

1. **Displacement of the respondents far away from health facilities**

- Yes
- No

1. **Fleeing of local health providers from a conflict zone**

- Yes
- No

1. **Looting or disruption of medical supplies to the health facilities**

- Yes
- No
- **Destruction of health facilities / Shutdown of health facilities**
- Yes
- No

4] Reason for not attending ANC visit:

- Clinic is far away
- No medicine in clinic
- No night duty staffs in clinic
- Clinic staff do not have good behavior
- Family does not allow
- No transport
- I have no money
- tobaccoThere is no one to accompany me.
